# Examining impulsivity as an endophenotype using a behavioral approach: a *DRD2 TaqI A *and *DRD4 48-bp VNTR *association study

**DOI:** 10.1186/1744-9081-3-2

**Published:** 2007-01-10

**Authors:** Dan TA Eisenberg, James MacKillop, Meera Modi, Joshua Beauchemin, David Dang, Stephen A Lisman, J Koji Lum, David S Wilson

**Affiliations:** 1 State University of New York at Binghamton, Biology Department, Binghamton, NY, USA; 2 State University of New York at Binghamton, Anthropology Department, Binghamton, NY, USA; 3 State University of New York at Binghamton, Psychology Department, Binghamton, NY, USA; 4 State University of New York at Binghamton, Laboratory of Evolutionary Anthropology and Health, Binghamton, NY, USA; 5 Center for Alcohol and Addiction Studies, Brown University, Providence, RI, USA

## Abstract

**Background:**

Research on the genetic basis for impulsivity has revealed an array of ambiguous findings. This may be a result of limitations to self-report assessments of impulsivity. Behavioral measures that assess more narrowly defined aspects of impulsivity may clarify genetic influences. This study examined the relationship between possession of the *DRD2 TaqI A *and *DRD4 48 bp VNTR *genetic polymorphisms and performance on a behavioral measure of impulsivity, the delay discounting task (DDT), and three traditional self-report measures.

**Methods:**

195 individuals (42% male) were recruited from a university campus and were assessed in small group sessions using personal computers. Genotyping was conducted using previously established protocols. For the *DRD2 TaqI A *locus, individuals were designated as possessing at least one copy of the A1 allele (A1+) or not (A1-), and for the *DRD4 48-bp VNTR *locus, individuals were designated as having at least one long allele (7 repeats or longer, L+) or not (L-). Principal analyses used multiple univariate factorial 2 (A1+/A1-) × 2 (L+/L-) analyses of variance.

**Results:**

A significant main effect of A1+ status on DDT performance was evident (*p *= .006) as well as a significant interaction effect (*p *= .006) between both genes. No other significant effects were evident on the self-report measures, with the exception of a trend toward an interaction effect on the Sensation Seeking Scale. Exploratory analyses suggested that the significant effects were not a function of population stratification or gender.

**Discussion:**

These data suggest that the *DRD2 TaqI A *and *DRD4 VNTR *polymorphisms influence impulsivity as measured with a delay discounting task. Specifically, these findings suggest that an interaction between the functional effects of the two unlinked genotypes results in significant difference in the balance of mesolimbic dopaminergic activation relative to frontal-parietal activation. However, these findings are also the first in this area and must be replicated.

**Conclusion:**

These findings suggest a meaningful interaction between the *DRD2 TaqI A *and *DRD4 VNTR *polymorphisms in the expression of impulsivity and provide initial support for the utility of using behavioral measures for clarifying genetic influences on impulsivity.

## Background

The effort to characterize the behavioral effects of genetic polymorphisms has produced a massive web of ambiguous associations and linkages [1-3]. One strategy to clarify the genetic bases of behavior is the endophenotype approach [2,4,5], which seeks to elucidate genetic associations with phenotypes of interest, typically diseases, by examining intermediary phenotypes (i.e., endophenotypes) that are more closely related to the functional influence of genetic variants. By characterizing endophenotypes, or "upstream" phenotypes that do not always result in the "downstream" disorder, progress may be made in both deconstructing the etiologies of complex psychiatric disorders and understanding the genetic and evolutionary basis for variation in non-disordered individuals [4]. In addition, endophenotypes are putatively more closely connected to genetic functionality, so larger magnitude genetic effects may be evident and thus more readily detectable in smaller samples [6,7], cf. [8].

Impulsivity is a prototypic candidate for the endophenotype approach because it is a trait that varies considerably in the overall population [9-12] and is associated with an array of psychiatric disorders. These include alcohol and drug dependence [13-19], pathological gambling [20,21], attention deficit-hyperactivity disorder (ADHD) [22,23], borderline personality disorder [24] and antisocial personality disorder [25-27]. Moreover, there is evidence for the heritability of impulsive behavior in both humans and non-human animals [28]. In terms of personality disorders, familial transmission of impulsive traits have been reported [24,29]. In addition, twin studies using the Karolinska Scale of Personality (KSP), Multidimensional Personality Questionnaire (MPQ) and Barratt Impulsivity Scale, Version 11 (BIS) also found substantial heritable components to impulsivity [30-33]. Similarly, impulsivity has also been demonstrated to be heritable in vervet monkeys as assessed by the Intruder Challenge Test [34], and in mice assessed by a delay discounting test [35]. However, impulsivity has also been found to vary with such factors as gender [36], age [37,38], education [37,39,40], health [39], savings [39] and parent rearing styles [41], suggesting that other variables also have a meaningful influence. Although the relative contributions of genetic and environmental variables are unclear at this point, converging lines of evidence suggest genetic factors play an important role.

As a result, a number of studies have explored the molecular genetic basis for variation in impulsivity by examining the associations between genetic polymorphisms and measures of impulsivity. Focusing on the serotonergic system, Preuss et al. [42] reported an association between A alleles of the *5HT2A *receptor – *G-1438A *polymorphism and increased impulsivity, but Patkar et al. [43] and Baca-Garceiro et al. [44] did not replicate that relationship. Within the dopamine system, Retz et al. [45] found an association between heterozygotes of the *DRD3 *single nucleotide polymorphism (SNP) and increased impulsivity, and Limosin et al. [46] found an association with the A2 alleles of the *DRD2 TaqI A *SNP and increased impulsivity in alcoholics, but both represent isolated reports. More broadly, in studies of the genetics of personality, impulsivity has been examined in the context of novelty seeking, a trait of which it is a cardinal feature [47]. From this perspective, a number of studies have found associations between long alleles of the *DRD4 48 bp Variable Number of Tandem Repeats *(*VNTR*) polymorphism and novelty-seeking, but many have not. One meta-analysis has found no overall association between *DRD4 48 bp *and novelty seeking [48], another a small effect [49] and a third review reports a positive association [50]. On balance, the current empirical literature is highly heterogeneous, in terms of the genes examined, phenotypic scales used and actual findings.

A limitation of the previous attempts to characterize genetic influences on impulsivity has been the prevailing reliance on self-report measures of impulsivity. There are a number of limitations to the self-report measures in general [51,52] and these apply also in the case of impulsivity. For example, individuals may vary considerably in their semantic construal of impulsivity-related question content and they may also vary in their positive or negative attributions about the content of the questions, creating an implicit or explicit response bias. Moreover, there is considerable evidence that individuals' self-reports can be substantially at variance with their actual behavior [51,52], suggesting that self-reported impulsivity may not always accurately reflect actual levels of impulsivity. This is further complicated by the fact that impulsivity is itself a multifaceted construct [28,53,54], including aspects of cognitive deliberation, reward valuation, behavioral inhibition and behavioral execution, among others. As such, it is unlikely that one genetic polymorphism would be pleiotropically responsible for all of these diverse facets, especially given that these different aspects are not always significantly associated with each other [e.g., [55,56]]. Indeed, there is ongoing debate as to which represent essential features of impulsivity, and which are different constructs altogether [e.g., [28,55,56]].

These limitations may be addressed by an increased emphasis on behavioral assessments of impulsivity. A number of behavioral indices of impulsivity have been developed [e.g., [57,58]] and these measures more objectively assess narrowly defined aspects of impulsive behavior and may reduce the bias of self-report. Moreover, in some cases, animal models and cognitive neuroscience approaches have illuminated the underlying neurobiology subserving behavioral performance on such measures [59-62], permitting more refined hypothesis testing of genetic variants that influence impulsivity. Although behavioral testing involves considerably greater experimental burden than self-report assessments, these measures may nonetheless substantially contribute to clarifying impulsivity as an endophenotype. These behavioral endophenotypes are expected to be more powerful than similar association studies which instead use broader psychological disorders as phenotypes.

The most widely studied behavioral measure of impulsivity is the delay discounting task (DDT). From a delay discounting perspective, impulsivity is defined as the relative preference for a smaller reward, sooner in time, compared to a larger reward, later in time [63]; that is, the amount a person discounts a reward based on its delay. Importantly, this measure of impulsivity has proven highly sensitive to increased impulsivity in psychiatric populations. More precipitous discounting (i.e., increased impulsivity) is associated with alcohol misuse [13,64,65], tobacco dependence [66,67], opiate dependence [68,69], stimulant dependence [70], pathological gambling [20,55,71], and antisocial personality disorder [27]. In addition, the DDT has been demonstrated to be stable over time [72].

Versions of the delay discounting paradigm may also be used to study impulsivity in animal models [35,59,60,73]. Neurobiologically, non-human research suggests that corticostriatal-mesolimbic substrates mediate delay discounting performance [59,61] and that dopamine is the critical neurotransmitter involved [60,73-75]. In addition, recent human neuroimaging findings indicate that preference for smaller immediate rewards is associated with greater mesolimbic activation, whereas preference for delayed rewards is associated with greater frontal-parietal activation [62]. Taken together, these findings suggest that impulsive decision-making from a delay-discounting perspective reflects a dynamic balance of frontal versus limbic dopaminergic activation. Importantly, there is indirect evidence that impulsivity as measured by delay discounting is heritable in humans [13] and direct evidence of its heritability in mouse strains [35].

Given the limitations to the current literature on impulsivity as an endophenotype and the potential promise of using behavioral measures, in the current study we examined impulsivity as a potential endophenotype using two dopaminergic genetic polymorphisms as candidates for observed variation in impulsivity as measured by the DDT and three traditional measures of impulsivity. These three measures include the BIS, Eysenck Impulsivity Questionnaire (EIQ), and the Sensation Seeking Scale – Form A (SSS), all of which have undergone extensive psychometric validation [11,12,76]. The BIS and EIQ are highly correlated and theoretically related scales, however they are associated with different neural activation profiles in a behavioral inhibition task [77], suggesting that they assess distinct facets of impulsivity. As previously mentioned, the BIS has shown a strong heritable component in a twin study [31]. Sensation seeking is a related construct to impulsivity, and has been shown to be both heritable and to potentially share genetically-mediated common biological mechanisms with impulsivity [33]. Additionally, SSS subscores are inversely related to KPS monotony avoidance, which has also been shown to be heritable [32]. In general, the empirical literature suggests performance on these measures is heritable, although this is clearly not definitive.

The two dopaminergic genetic polymorphisms we examined were the *DRD2 TaqI A *and *DRD4 48 bp VNTR *polymorphisms. Both have been associated with psychiatric disorders involving impulsivity, namely substance abuse and ADHD (for reviews, see [78,79]). In addition, the two polymorphisms appear to functionally influence the dopamine D_2 _and D_4 _receptors, which are densely located in the corticostriatal-mesolimbic system [80-87], the apparent neurobiological substrate and neurotransmitter system underlying delay discounting [59-61,73-75].

The *DRD2 TaqI A *site is a SNP with two possible alleles, the major A2, and minor A1. The A1+ genotype (heterozygous or homozygous A1) has been most strongly associated with substance abuse, particularly alcoholism [83], albeit with some controversy. The A1+ genotype has also been related to pathological gambling, novelty seeking, and sensation seeking [88]. The *DRD2 TaqI A *site is 9.4 kb downstream from the coding region for the dopamine D_2 _receptor gene. It is not in any known regulatory region, and although the A1 allele is associated with a decrease in dopamine D_2 _binding and glucose metabolic rates in many brain regions [83,89,90], its mechanism for influencing DRD2 expression is unknown. The *TaqI A *polymorphism is also located in a nearby kinase gene, the *Ankyrin Repeat and Kinase Domain Containing 1 (ANKK1) *gene, where it causes a Glutamate→ Lysine substitution [91,92]. The results of the amino acid substitution are not known, but could impact interactions of ANKK1 proteins with other proteins including the dopamine D_2 _receptor [92]. No other polymorphism has been revealed in linkage disequilibrium with *TaqI *A that could easily account for these associations [91-93].

The *DRD4 48-bp VNTR *polymorphism is in exon 3 of the gene coding for the dopamine D_4 _receptor. The *VNTR *polymorphism varies between 2 and 11 repeats of a similar 48 bp coding region sequence, with a trimodal distribution of 2, 4 and 7 repeat alleles (2R, 4R and 7R) in most, but not all, populations [94]. Although the functional significance of the *DRD4 VNTR *polymorphism has not been definitively characterized, long alleles (typically 7R as opposed to 4R) have been generally found to be functionally less reactive in in-vitro expression experiments [95-99], with some heterogeneity [100-104]. Additionally, in-vivo pharmacological treatments are also generally consistent with 7R alleles resulting in less responsive D_4 _receptors than 4R alleles [105-109].

We predicted that possession of at least one A1 allele for the *DRD2 TaqI A *and at least one long allele (7-repeats or longer) of the *DRD4 VNTR *genotype would be associated with greater impulsivity. Moreover, we predicted that the delay discounting task would be more sensitive than the self-report measures, as reflected in larger magnitude effects. However, we did not predict one polymorphism to be more likely to exhibit significant associations relative to the other. Finally, based on previous findings reporting interactions between D_2 _and D_4 _receptor genes [e.g., [110]], we also examined both potential interactive effects (i.e., quantitatively disproportionate effects based on a combination of polymorphisms of both genes) and potential additive effects (i.e., linearly increasing effects based on a combination of polymorphisms of both genes).

## Methods

### Participants

A total of 195 unselected subjects were recruited from the Human Subject Research Pool at the State University of New York at Binghamton and are described demographically in Table 1. Because population stratification is a potential problem in genetic association studies [111,112], racial ancestry was closely examined by asking participants to identify the ancestry of all four of their grandparents, following the recommendation of Shields et al. [113]. Participants were allowed to select as few or as many ancestry groups to describe each grandparent from the following categories: European, African American, East Asian, South Asian, Middle Eastern, Native North American, Native South American, Pacific Islander, African and an open ended "other" category. Based upon several respondents identifying grandparents as Hispanic, Latino, Puerto Rican, and Caribbean in the "Other" category, a group termed "Latin American" was constituted, including those groups and Native South Americans.

**Table 1:** Demographic information

**Variable**	**Descriptive Statistics**
Sex	42% male; 58% female
Age	Median = 19.33 (IQR = 18.83–20.35)
Household Income	Median = $85,000 (IQR = $50,000 – 120,000)
Ethnicity	44.1% European, 14.4% East Asian, 11.8% Latin American, 5.1% South Asian, 3.1% Native North American, 1.5% African American, 1.0% Pacific Islander, 1.0% African, 13.8% multiracial, 5.6% unknown. (does not sum to exactly 100.0% because of rounding)

Subject Characteristics (*n *= 195).

### Genotyping

DNA was collected with QuickExtract buccal swabs and extracted with BuccalAmp solution as directed by the manufacturer (Epicenter). Subjects were instructed to rinse their mouths out with water before swabbing. *DRD2 TaqI A *was typed with a PCR/RFLP method [based on [114]; see Additional File 1]. *DRD2 TaqI A *allele frequencies and genotypes are presented in Table 2, and are distributed in the sample population in Hardy-Weinberg (HW) equilibrium (Fisher's Exact Test, *p *= 1.0). The *DRD4 VNTR *locus was genotyped using an adaptation of a previous protocol [[115]; see Additional File 1]. *DRD4 VNTR *allele frequencies and genotypes are presented in Table 3, and were in HW equilibrium (Markov Chain algorithm, *p *= 0.38).

**Table 2:** *DRD2 TaqI A *allele frequencies, genotypes and genotype classifications

**Allele/Genotype**	**n**	**%**
Allele		
A2	274	70.3
A1	116	29.7
Total	390	100.0
Genotype		
A2/A2	96	49.2
A2/A1	82	42.1
A1/A1	17	8.7
Total	195	100.0
Genotype Classification*		
A1+	99	50.8
A1-	96	49.2
Total	195	100.0

*A1+ subjects have at least one A1 allele and A1- subjects are homozygous for A2.

**Table 3:** *DRD4 VNTR *allele frequencies, genotypes and genotype classifications

**Allele/Genotype**	**n**	**%**
Allele		
2	39	10.1
3	15	3.9
4	265	68.7
5	1	0.3
7	64	16.6
9	2	0.5
Total	386	100.0
Genotype		
2/2	3	1.6
2/3	1	0.5
2/4	30	15.5
2/7	2	1.0
3/3	1	0.5
3/4	10	5.2
3/7	2	1.0
4/4	85	44.0
4/5	1	0.5
4/7	52	26.9
4/9	2	1.0
7/7	4	2.1
Total	193	100.0
Genotype Classification*		
L-	131	67.9
L+	62	32.1
Total	193	100.0

*L+ subjects have at least one allele 7 repeats or longer and L- subjects have both alleles shorter than 7 repeats.

### Delay discounting task

To capture delay discounting empirically, the DDT poses participants with repeated choices between a smaller reward received immediately and a greater reward received after some time delay (e.g., "Would you prefer to have $65 today or $100 in a month?"). Over the course of the task, the amounts of immediate rewards are successively modified, as is the duration of delay. The individual's responses to the entire array of choices are then used to empirically derive their discounting function (i.e., how steeply they discount delayed rewards relative to immediate rewards, commonly denoted *k*). The DDT was administered with hypothetical money via a custom computer program [[116]; see Additional File 2]. The temporal discounting function (*k*) was generated using Mazur's hyperbolic discounting equation [117]. Model fits of how well subjects discounting functions fit Mazur's equation were calculated as *R*^2 ^values. Erratic subjects and those with *R*^2 ^values below 0.30 were excluded from principal analyses [118].

### Self-report measures

The self report measures administered and analyzed here include the BIS, EIQ and SSS. The BIS provides an overall measure of impulsivity and three relevant subscores: Attentional Impulsiveness, Motor Impulsiveness and Non-Planning Impulsiveness [12]. The EIQ is a self-report measure of impulsivity that generates three subscales, of which two were relevant to the current study: Impulsiveness, and Venturesomeness [10]. The SSS provides an overall measure of sensation seeking proneness and four also relevant lower order factors: Experience Seeking, Boredom Susceptibility, Disinhibition, and Thrill and Adventure Seeking [76].

### Procedures

All procedures were approved by the Human Subjects Research Review Committee at the State University of New York at Binghamton and all subjects gave informed consent. Participants attended group assessment sessions (maximum = 10) and were initially provided with oral instructions followed by the DNA sample collection. Participants then completed the delay discounting task followed by the self-report measures administered in random order. In addition to the oral instructions, the DDT task and other measures all were accompanied by on-screen written instructions and experimenters (DTAE and/or MM) were available throughout the sessions for questions. The sessions lasted approximately one hour.

### Data analysis

Raw scores were calculated for all self-report scales and are described in Table 4. All data were examined for outlying data points, distribution normality, and missing data. The most skewed non-normal scale was the DDT before log transformation, but log transformation provided adequate correction. To assure missing responses were not systematically biased by genotype, missing versus non-missing data for each psychometric scale was analyzed across both *DRD2 TaqI A *and *DRD4 VNTR *genotypes via 2 × 2 contingency tables. Missing values were not imputed, but were excluded from analysis. HW equilibria were tested with the HWE program [119]. *DRD2 *HW equilibrium was tested with Fisher's Exact and *DRD4 *was tested with the Markov Chain algorithm. Based on previous association studies, for the *DRD2 TaqI A*, individuals with at least one A1 allele were designated as A1+ and those who were homozygous for the A2 allele were designated A1-. Similarly, DRD4 VNTR genotypes were separated into long allele (7 repeats or longer) present (L+) and long allele absent (L-) groups. The principal analyses used 2 (A1+/A1-) × 2 (L+/L-) factorial analyses of variance (ANOVAs), using a two-tailed significance criterion of *p *< .05 and partial eta squared (η_p_^2^) as a measure of effect size. Multiple factorial ANOVAs were conducted rather than a multivariate approach based on the study's premise that more narrowly conserved facets of impulsivity would be more sensitive to allelic variation. Where applicable, potential additive effects were examined by conducting post-hoc one-way ANOVAs.

**Table 4:** Descriptive statistics

	**N**	**Mean**	**Min**	**Max**	**SD**
**BIS Attentional Impulsivity**	182	17.03	9	29	3.49
**BIS Motor Impulsivity**	185	21.68	13	34	3.73
**BIS Non-planning Impulsivity**	181	25.38	14	38	4.07
**BIS Total**	168	63.93	37	95	9.24
**EIQ Ventursomeness**	190	9.58	0	17	3.51
**EIQ Impulsivity**	182	10.11	0	22	4.44
**SSS Disinhibition**	185	5.63	0	10	2.53
**SSS Experience Seeking**	186	5.40	0	10	1.96
**SSS Boredom Susceptibility**	190	3.07	0	8	2.02
**SSS Thrill & Adventure Seeking**	190	6.48	0	10	2.75
**SSS Total**	179	20.57	6	34	6.09
DDT*	166	0.05	0.00	1.72	0.02–0.17

* untransformed DDT value with median and IQR given instead of Mean and SD

BIS = Barratt impulsivity Scale; EIQ = Eysenck Impulsivity Questionnaire; SSS = Sensation Seeking Scale; DDT = Delay Discounting Task.

Several exploratory analyses were employed to examine potential alternative influences. To examine the potential for population stratification we used a two-step process. First, for variables where significant effects were detected, one-way between-subjects ANOVAs were conducted to determine whether any significant differences by ethnic group were evident using only subjects without known admixture who were part of groups making up at least 10% of the sample. Second, significant results were re-examined within the largest single racial group (i.e., a group with minimized racial admixture), in this case, the European ancestry group. Participants for this analysis were weighted by the percentage of European ancestry reported. Rather than emphasizing statistical significance based on F-ratio, due to the inherently diminished power by reduced sample size, comparisons were made between effect sizes in the findings from the overall sample and the subsample. In addition to exploratory analyses based on racial ancestry, because similar studies have found notable genotype by gender interaction effects [e.g., [88,120]], follow-up exploratory analyses were conducted to examine gender influences using 2 (A1+/A1-) × 2 (L+/L-) × 2 (Male/Female) factorial ANOVAs on the independent variables. In addition, because a fit criterion was used for DDT performance, exploratory analyses were also conducted using all *k *values. Finally, 2 (*DRD4*) × 2 (A1+/A1-) analyses were conducted again with alternative methods of parsing *DRD4 *genotypes [see Additional File 3]. Corrections for multiple tests were not employed because of the exploratory and explicitly comparative nature of our analysis [121], and because with correlated dependent variables, such corrections would be too conservative [122].

## Results

### Initial analyses

**Figure 1:**
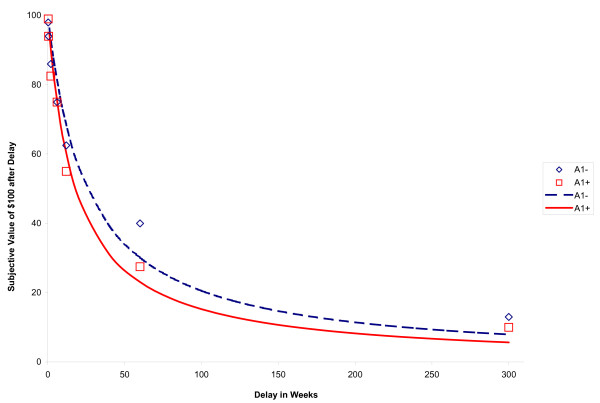
**Main effect of *DRD2 Taq*I *A *on delay discounting**. Subjective value of $100 from one week to twenty-five years for A1+ and A1- individuals. Squares show the median points of indifference for A1+; diamonds show the median points of indifference for A1- subjects. The hyperbolic curves derived from the median k values of A1+ (continuous line) and A1- groups (dotted line) are given.

**Figure 2:**
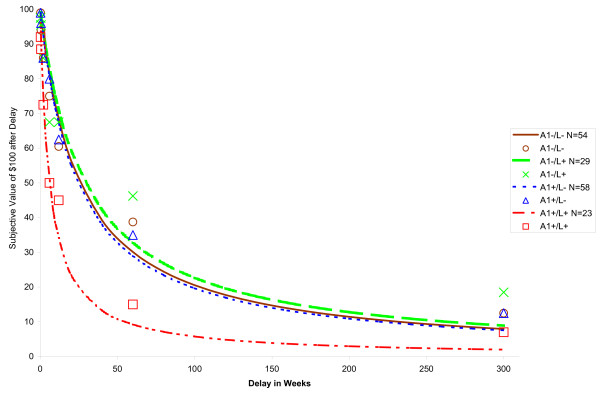
***DRD2 *by *DRD4 *interaction effect on delay discounting**. Subjective value of $100 from one week to twenty-five years for individuals with each allelic combination. Median points of indifference of the four groups are provided with derived hyperbolic curves.

Of the total sample of 195 subjects, 8 were clearly uncooperative on the DDT and provided erratic responses, resulting in exclusion from subsequent analysis. Of the remaining 187 subjects, Mazur's [117] equation provided a good fit overall, typically accounting for over 90% of the variance (median *R*^2 ^= 0.904, interquartile range [IQR] = .72 – .96), which is comparable to past studies [9,123]. The fit to Mazur's equation did not differ by genotype (Factorial ANOVA 2 [A1+/A1-] × 2 [L+/L-]; results not shown). However, an additional 21 subjects did not meet the model fit criterion (*R*^2 ^≤ .30), and were excluded from consideration in the primary DDT analyses. This further increased the median *R*^2 ^value for the remaining 166 subjects (*R*^2 ^= .92, IQR = .82 – .97). As anticipated, subjects' performance on the DDT topographically resulted in hyperbolic discounting curves, exhibiting precipitous initial discounting followed by more modest decreases based on delay (Figures 1 and 2).

Between 2.6% and 13.8% of values were missing from each of the remaining self-report scales because subjects skipped one or more items or incorrectly entered their subject ID number (Table 4). There were no significant biases in missing value distributions by *DRD2 *genotype, although for *DRD4*, L- subjects were missing more data (7.6%) than L+ (0%) for only the BIS Motor Impulsivity scale (Fishers Exact *p *= .032). Pearson's product-moment correlations between the indices of impulsivity are shown in Table 5. Consistent with previous research, many significant associations were evident, ranging from modest to high magnitudes. As would be expected, the highest magnitude associations were between measure totals and subscales of the same measure, due to the redundancy of items.

**Table 5:** Pearson's product moment correlations between dependent variables.

	**Variable**	**1**	**2**	**3**	**4**	**5**	**6**	**7**	**8**	**9**	**10**	**11**
**1**	**BIS Attentional Impulsivity**											
**2**	**BIS Motor Impulsivity**	0.542**										
**3**	**BIS Non-planning Impulsivity**	0.447**	0.486**									
**4**	**BIS Total**	0.804**	0.836**	0.813**								
**5**	**EIQ Ventursomeness**	0.110	0.244**	0.112	0.185*							
**6**	**EIQ Impulsivity**	0.531**	0.559**	0.442*	0.634**	0.245**						
**7**	**SSS Disinhibition**	0.298**	0.337**	0.234**	0.350**	0.240*	0.425**					
**8**	**SSS Experience Seeking**	0.119	0.199**	0.028	0.123	0.455**	0.231**	0.187*				
**9**	**SSS Boredom Susceptibility**	0.307**	0.359**	0.282**	0.386**	0.302**	0.515**	0.437**	0.275**			
**10**	**SSS Thrill & Adventure Seeking**	0.018	0.194**	0.064	0.114	0.799**	0.048	0.147*	0.368**	0.102		
**11**	**SSS Total**	0.281**	0.412**	0.232**	0.377**	0.692**	0.440**	0.681**	0.649**	0.644**	0.660**	
**12**	**DDT**	0.08	0.133*	0.124	0.15	0.008	0.205*	0.119	-0.015	0.069	-0.038	0.041

BIS and SSS Total Scores are made up of the sum of their respective subscores, explaining those high correlations. **p < .01, *p < .05 (two-tailed)

### Influences of DRD2  TaqI A and DRD4 VNTR genotypes on impulsivity

Factorial 2 (*DRD2 *A1+/A1-) × 2 (*DRD4 *L+/L-) ANOVAs were run for each impulsivity index. There was a main effect of *DRD2 TaqI A *genotype (*F (*1,164) = 7.65, *p *= .006, η_p_^2 ^= .046), reflecting greater delay discounting measured impulsivity in A1+ individuals, and an interaction effect between the two genotypes (*F *(1, 164) = 7.63, *p *= .006, η_p_^2 ^= .046), such that A1+/7R+ individuals exhibited the greatest delay discounting. Both effects are indicated in Table 6. Figures 1 and 2 show the derived hyperbolic discounting functions for each genotype and the median POI values for each delay interval. Follow-up one-way ANOVAs based on genotype combinations revealed that A1+/7R+ individuals exhibited significantly steeper discounting curves from all other combinations (*p*s = .001–.007), but the other three groups were not significantly different from each other (*p*s = .241 – .968).

**Table 6:** Performance on Measures of Impulsivity by Genotype and Significance values

	**DRD2**					**DRD4**					**Interaction**
	
	**A1+**		**A1-**		**p**	**L+**		**L-**		**p**	**p**
	**Mean**	**SE**	**Mean**	**SE**		**Mean**	**SE**	**Mean**	**SE**		
**BIS Attentional Impulsivity**	16.49	0.41	17.58	0.32	0.171	17.12	0.50	16.92	0.2975	0.791	0.223
**BIS Motor Impulsivity**	21.19	0.39	22.17	0.38	0.131	21.68	0.49	21.59	0.3311	0.933	0.510
**BIS Non-planning Impulsivity**	25.22	0.46	25.56	0.40	0.783	25.08	0.46	25.38	0.3807	0.636	0.660
**BIS Total**	62.80	1.08	65.14	0.93	0.261	63.79	1.15	63.65	0.8826	0.999	0.289
**EIQ Ventursomeness**	9.63	0.37	9.53	0.35	0.642	9.56	0.39	9.62	0.3316	0.945	0.664
**EIQ Impulsivity**	9.70	0.50	10.51	0.42	0.469	10.20	0.57	9.97	0.4013	0.741	0.347
**SSS Disinhibition**	5.40	0.27	5.86	0.25	0.583	5.81	0.32	5.52	0.2295	0.487	0.269
**SSS Experience Seeking**	5.44	0.21	5.36	0.20	0.360	5.71	0.25	5.25	0.1774	0.130	0.134
**SSS Boredom Susceptibility**	3.15	0.20	2.99	0.21	0.227	3.27	0.27	2.98	0.1754	0.314	0.193
**SSS Thrill & Adventure Seeking**	6.54	0.29	6.43	0.27	0.603	6.20	0.34	6.64	0.2495	0.346	0.514
**SSS Total**	20.63	0.66	20.51	0.63	0.323	20.77	0.74	20.48	0.5745	0.744	*0.062*
DDT*	0.06	.02–.21	0.04	.01–.15	**0.006**	0.08	.02–.19	0.04	.02–.14	0.183	**0.006**

*untransformed DDT value with median and IQR given instead of Mean and SE, p values from log transformed values

BIS = Barratt impulsivity Scale; EIQ = Eysenck Impulsivity Questionnaire; SSS = Sensation Seeking Scale; DDT = Delay Discounting Task.

No significant effects were evident for any of the self-report measures of impulsivity, although a marginally significant interaction effect of *DRD2TaqI A *and *DRD4 VNTR *was evident on the SSS Total scale (*F *(1,177) = 3.52, *p *= .06, η_p_^2 ^= .02). This effect also reflected greater sensation seeking in the A1+/L+ group, and is depicted in Table 6.

### Exploratory analyses

We explored the possibility of population stratification using the proposed approach. A one-way ANOVA revealed no significant effect of racial group on DDT *k *values (*F *(1,116) = 1.556, *p *= .215, η_p_^2 ^= .027). Median k values (IQR) across groups accounting for at least 10% of the sample were: European, .0416 (.0140–.1600); East Asian, .0349 (.0112–.1505), and Latin American, .0727 (.0133–.2198). Repeating the principal analyses in the largest single racial group, those with European ancestry, the main effect of *DRD2 *on DDT *k *values was marginally significant (*F *(1,74) = 3.487, *p *= .066, η_p_^2 ^= .047), as was the interaction effect of *DRD2 *and *DRD4 *on DDT k (*F *(1,74) = 2.729, *p *= .103, η_p_^2 ^= .038). However, the η_p_^2 ^effect sizes were very similar between the analysis of all subjects and European ancestry subsample, suggesting that the reduction of statistical significance was related to reduced statistical power in the smaller subsample. Taken together, these analyses suggest that the significant effects were not the result of population stratification.

Potential gender effects were examined by including gender as an additional two-level independent variable, finding several significant main gender effects (BIS Motor Impulsivity: *F (*1,183) = 13.448, *p *< .001, η_p_^2 ^= .071; BIS Total *F *(1,166) = 6.570, *p *= .011, η_p_^2 ^= .040; EIQ Venturesomness: *F (*1,188) = 8.924, *p *= .003, η_p_^2 ^= .047; EIQ Impulsivity: *F *(1,180) = 4.499, *p *= .035, η_p_^2 ^= .025; SSS Boredom Susceptibility: *F (*1,188) = 4.731, *p *= .038, η_p_^2 ^= .024) with males scoring higher in each case, which was not an unexpected sexual dimorphism [36,124]. The *DRD2 TaqI A *effect and interaction effect on DDT performance remained significant with the inclusion of gender as an independent variable (*DRD2 TaqI A*: *F *(1,164) = 8.585, *p *= .004, η_p_^2 ^= .052; Interaction: *F *(1,164) = 7.683, *p *= .006, η_p_^2 ^= .047), further bolstering the stability of the finding. All other main and interaction effects were non-significant (not shown). Finally, to explore whether the use of a model fit criterion affected the DDT findings, the data were re-analyzed using all *k *values. This resulted in the same *DRD2 TaqI *A main effect (*DRD2*: *F *(1,183) = 9.668, *p *= .002 η_p_^2 ^= .051) and interaction effect (*F (*1,183) = 4.830, *p *= .029, η_p_^2 ^= .026).

Using a Factorial ANOVAs with *DRD2 *by *DRD4*, three additional methods of parsing DRD4 48 bp VNTR genotypes yielded similar results to the primary method used above [see Additional File 3].

## Discussion

The premises of this study were that the ambiguity in understanding genetic contributions to impulsivity may be due to limitations of self-report measures and that behavioral tasks measuring discrete facets of impulsivity may address this issue. This hypothesis was broadly supported by the results. Performance on the DDT revealed a significant main effect of *DRD2 TaqI A *status, such that A1+ individuals exhibited greater impulsivity, and a significant *DRD2 TaqI A *by *DRD4 48 bp VNTR *interaction, such that possession of at least one A1+ allele and at least one L+ allele was associated with a pronounced increase in impulsivity. These differences in delay discounting were quite dramatic; for example, A1+/L+ subjects valued $100 less than half as much as all the other allelic combinations at a 5-year delay (Figure 2). Importantly, these relationships did not appear to be a function of population stratification or gender.

Beyond delay discounting, it was notable that there were no significant genetic associations evident for any of the survey-based self-report measures, with the exception of a trend toward greater sensation seeking reflecting the same *DRD2 TaqI A *by *DRD4 VNTR *interaction. This could be because the survey-based scales generated scores that were based on the individual's subjective perception of a variety of distal behavioral tendencies and the relationships were muddied by self-report biases. Alternatively, it is possible that the facets of impulsivity assessed by the survey-based measures are simply not relevant endophenotypes of the genes examined in this study. Consistent with evidence of impulsivity as a multifaceted construct [12,53], mixed correlations were evident across the various measures (Table 5), revealing moderate associations among the self-report measures, but generally negligible associations between the DDT and the other indices. Previous studies have reported both significant and nonsignificant correlations between delay discounting and self-report measures of impulsivity [65,68,125-127]. The overlap between these different facets and methods of assessing impulsivity appears to be variable by sample.

There are a number of aspects of these findings that warrant further discussion, particularly the delay discounting findings. The *DRD2 TaqI A *main effect was consistent with our hypotheses, but we also predicted a main effect for *DRD4 VNTR *genotype, which was not evident, and an interaction between the two was examined based on its plausibility, but was not predicted a priori. In terms of the *DRD2 TaqI A *main effect, subsequent analyses revealed that the most meaningful role of A1+/A1- status was actually in combination with *DRD4 VNTR *genotype in an interaction effect on performance. However, in understanding this effect, an important distinction must be made between additive and interactive genetic effects. In this case, an additive effect would refer to possession of each allele of interest being associated with greater impulsivity, and possession of alleles at both loci being associated with a linearly greater level of impulsivity. A clear example of an additive genetic effect is exemplified in two recent studies [128,129], in which polymorphisms of the DRD2 *TaqI A *and *SLC6A3 *genes were examined in reference to stress- and cue-elicited craving in African-American smokers. Both polymorphisms of interest were associated with greater craving, and exhibited an additive effect such that possession of neither polymorphism was associated with the least amount of craving, possession of either was associated with greater craving, and possession of both was associated with the greatest amount of craving.

In contrast, an interactive effect in this case would refer to the case in which possession of the two alleles would be associated with quantitatively disproportionate level of impulsivity, which was the case in the current study. Individuals who were A1+/L+ exhibited disproportionately more precipitous delay discounting than all other groups, whereas all other allelic combinations exhibited highly similar levels of discounting (Figure 2). An additive effect, which would have been evident if both polymorphisms of interest exhibited main effects and a linear increase in performance based on the possession of both candidate alleles, was not evident.

With regard to the mechanisms of underlying the interaction between the two loci, any discussion must be speculative given that the two polymorphisms under consideration have not been definitively characterized. Acknowledging this ambiguity, the observed associations may be understood in the context of the relative neuroanatomical localization of the D_2 _and D_4 _receptors. Dopamine D_2 _receptors seem to play a more prominent role in the striatum than D_4 _receptors, whereas D_4 _receptors seem to be more influential in the prefrontal cortex and cortex than D_2 _receptors [80, 84, 85, 86, 87, 130, 131, 132, 133, 134], however: [135, 136]. As observed with functional magnetic resonance imaging (fMRI), choices for immediate smaller rewards is associated with greater peak activation in the ventral striatum and regions of the medial prefrontal cortex and less relative activation in regions of the lateral prefrontal and parietal cortex [62]. In contrast, for choices for delayed rewards, the relative activations are reversed, with generally greater lateral prefrontal and parietal activation and decreased activation of the ventral striatum and medial prefrontal cortex [62]. Other studies suggest more generally that behavioral inhibition, a fundamental part of impulsivity is modulated via dopamine systems in the striatum and prefrontal cortex [28]. Together, this suggests that the D_2 _polymorphism may be modifying more primitive limbic neural systems involved in reward salience while the D_4 _polymorphism may be modifying higher-level systems involved in abstract thinking, deliberation and behavioral inhibition. Thus, particularly steep delay discounting (increased impulsivity) in those with both long *DRD4 *and A1 *DRD2 *alleles may reflect the concurrent variation in two key points in the reward decision-making loops spanning the corticostriatal-mesolimbic axis. Specifically, the pattern of findings suggest that for those individuals who have a greater sensitivity to reward based on possession of the *DRD2 A1 *allele, decreased frontal-cortical inhibition resulting from possession of a *DRD4 VNTR *long allele results in substantially greater discounting of delayed rewards.

Of interest, however, the contrapositive relationship by genotype was not indicated. Considered together, the presence of a *DRD2 TaqI A *main effect, the absence of a *DRD4 VNTR *main effect, and a significant interaction suggests that the influence of A1+-mediated functional variation in striatal D_2 _receptors has primacy over the influence of L+-mediated cortical D_4 _receptor variation in modulating inter-temporal choice. That is, for individuals with a mesolimbic dopamine system that is predisposed to favor dopaiminergic rewards (A1+), the presence of an allele that may be associated with reduced frontal cortical inhibitory control (L+) may result in disproportionately greater discounting of delayed rewards, but not the other way around. In cognitive terms, the results suggest that the effects of inhibitory considerations of the future are superimposed on the limbic signals of the incentive salience of immediate rewards. However, we reiterate that given that the functional roles of both genetic polymorphisms are far from fully understood, this explanation must be speculative at this point. Future studies using neuroimaging techniques may directly address this interpretation.

Assuming these findings are genuine and can be replicated, they have potential implications for the ambiguity in association studies of genetic variables in psychiatric disorders [1-3]. To date, single locus association studies have typically reported mixed findings of significant associations and failures to replicate. The current data, and other studies revealing interactions between unlinked loci [e.g., [110, 137, 138], suggest that single gene association studies may overlook the fact that important facets of a disorder may depend not only on independent effects of polymorphism, but also on interactions between multiple genes within a given system.

There are a number of qualifications of these findings worth noting. Although they provide preliminary support for the notion that behavioral aspects of impulsivity may be more amenable to investigations of genetic influences, to our knowledge they represent the first report of specific genetic influences on delay discounting. As such, they must be replicated to affirm their empirical validity and to more conclusively affirm the potential of a task-based approach to generate more reliable findings than self-report measures. In addition, it should be noted that these findings most clearly apply to the European ancestry subsample, which both represented the largest proportion of subjects and was sufficiently large to affirm the findings independent of other ethnic subsamples. As such, although we found no evidence of population stratification, studies of delay discounting as a potential endophenotype in larger samples of non-European descendants will be important to address the generalizability of these findings.

## Conclusion

In summary, the current study sought to examine the influences of two genetic polymorphisms on impulsivity using a behavioral task and traditional self-report measures. The findings indicated both independent and interactive genetic effects on the behavioral task, suggesting that genetically-based functional differences in the corticostriatal-mesolimbic dopamine system are responsible for variation in impulsivity from a delay discounting perspective. Rather than being a function of a single locus, these data suggest that the most prominent effect was as result of an interaction between polymorphisms of the *DRD2 TaqI A *and *DRD4 VNTR *genes. Although these findings must be replicated, they may contribute to understanding the genetic basis of impulsivity and provide support for the notion of using behavioral tasks in doing so.

## Authors' contributions

DTAE participated in genotyping and phenotyping of subjects, study design, coordination, statistical analysis, and drafting the manuscript.

JM conceived the study, participated in study design, coordination, statistical analysis, and drafting the manuscript.

MM participated in genotyping and phenotyping of subjects and study design and coordination.

JB participated in genotyping

DD participated in genotyping

SAL participated in study design.

JKL oversaw genotyping, and participated in study design and coordination.

DSW oversaw the study design and coordination.

## Supplementary Material

Additional File 1 *DRD2 TaqI A *and *DRD4 48 bp VNTR *genotyping protocols Click here for file

Additional File 2 Delay discounting protocol Click here for file

Additional File 3 **Alternative DRD4 parsing method influences on DRD2 × DRD4 ANOVA to predict impulsivity phenotypes**. This supplement explores parsing DRD4 using three alternative methods, and finds similar results to that of the main manuscript. Click here for file

## References

[B1] Lucentini (2004). Gene association studies typically wrong. Scientist.

[B2] Munafo MR (2006). Candidate gene studies in the 21st century: meta-analysis, mediation, moderation. Genes Brain Behav.

[B3] Munafo MR, Clark TG, Moore LR, Payne E, Walton R, Flint J (2003). Genetic polymorphisms and personality in healthy adults: A systematic review and meta-analysis. Mol Psychiatry.

[B4] Gottsman II, Gould TD (2003). The Endophenotype Concept of Psychiatry: Etymology and Strategic Intentions. Am J Psychiatry.

[B5] Berrettini WH (2005). Genetic bases for endophenotypes in psychiatric disorders. Dialogues Clin Neurosci.

[B6] Freimer N, Sabatti C (2004). The use of pedigree, sib-pair and association studies of common diseases for genetic mapping and epidemiology. Nat Genet.

[B7] Freimer N, Sabatti C (2003). The Human Phenome Project. Nat Genet.

[B8] Flint J, Munafo MR (2006). The endophenotype concept in psychiatric genetics. Psychol Med.

[B9] Green L, Myerson J (2004). A discounting framework for choice with delayed and probabilistic rewards. Psychol Bull.

[B10] Eysenck SBG, Eysenck HJ (1978). Impulsiveness and Venturesomeness - Their Position in a Dimensional System of Personality Description. Psychol Rep.

[B11] Eysenck SBG, Pearson PR, Easting G, Allsopp JF (1985). Age Norms for Impulsiveness, Venturesomeness and Empathy in Adults. Pers Individ Dif.

[B12] Patton JH, Stanford MS, Barratt ES (1995). Factor Structure of the Barratt Impulsiveness Scale. J Clin Psychol.

[B13] Petry NM, Kirby KN, Kranzler HR (2002). Effects of gender and family history of alcohol dependence on a behavioral task of impulsivity in healthy subjects. J Stud Alcohol.

[B14] Colder CR, Chassin L (1997). Affectivity and impulsivity: Temperament risk for adolescent alcohol involvement. Psychol Addict Behav.

[B15] Devieux J, Malow R, Stein JA, Jennings TE, Lucenko BA, Averhart C, Kalichman S (2002). Impulsivity and HIV risk among adjudicated alcohol- and other drug-abusing adolescent offenders. Aids Educ Prev.

[B16] Labouvie EW, McGee CR (1986). Relation of Personality to Alcohol and Drug-Use in Adolescence. J Consult Clin Psychol.

[B17] Nagoshi CT, Wilson JR, Rodriguez LA (1991). Impulsivity, Sensation Seeking, and Behavioral and Emotional Responses to Alcohol. Alcohol Clin Exp Res.

[B18] Grano N, Virtanen M, Vahtera J, Elovainio M, Kivimaki M (2004). Impulsivity as a predictor of smoking and alcohol consumption. Pers Individ Dif.

[B19] Geist CR, Herman SM (1990). A comparison of the characteristics of smokers, ex-smokers, and non-smokers. J Clin Psychol.

[B20] MacKillop J, Anderson EJ, Castelda B, Mattson R, Donovick P Divergent validity of self-report and behavioral assessment measures in pathological gamblers. J Gambl Stud.

[B21] Steel Z, Blaszczynski A (1998). Impulsivity, personality disorders and pathological gambling severity. Addiction.

[B22] American Psychiatric Association (1994). Diagnostic and Statistical Manual of Mental DISORDERS, DSM-IV, 4th edn..

[B23] Sagvolden T, Johansen EB, Aase H, Russell VA (2005). A dynamic developmental theory of attention-deficit/hyperactivity disorder (ADHD) predominantly hyperactive/impulsive and combined subtypes. Behav Brain Sci.

[B24] Siever LJ, Torgersen S, Gunderson JG, Livesley WJ, Kendler KS (2002). The borderline diagnosis III: Identifying endophenotypes for genetic studies. Biol Psychiatry.

[B25] Fossati A, Barratt ES, Carretta I, Leonardi B, Grazioli F, Maffei C (2004). Predicting borderline and antisocial personality disorder features in nonclinical subjects using measures of impulsivity and aggressiveness. Psychiatry Res.

[B26] Luengo MA, Carrillodelapena MT, Otero JM, Romero E (1994). A Short-Term Longitudinal-Study of Impulsivity and Antisocial-Behavior. J Pers Soc Psychol.

[B27] Petry NM (2002). Discounting of delayed rewards in substance abusers: relationship to antisocial personality disorder. Psychopharmacology.

[B28] Congdon E, Canli T (2005). The Endophenotype of Impulsivity: Reaching Consilience Through Behavioral, Genetic, and Neuroimaging Approaches. Behav Cogn Neurosci Rev.

[B29] Silverman JM, Pinkham L, Horvath TB, Coccaro EF, Klar H, Schear S, Apter S, Davidson M, Mohs RC, Siever LJ (1991). Affective and Impulsive Personality-Disorder Traits in the Relatives of Patients with Borderline Personality-Disorder. Am J Psychiatry.

[B30] Pedersen NL, Plomin R, McClearn GE, Friberg L (1988). Neuroticism, extraversion and related traits in adult twins reared apart and reared together. J Pers Soc Psychol.

[B31] Seroczynski AD, Bergeman CS, Coccaro EF (1999). Etiology of the impulsivity aggression relationship: Genes or environment?. Psychiatry Res.

[B32] Saudino KJG (1999). Genetic and Environmental Influences on Personality in Adult Russian Twins. Int J Behav Dev.

[B33] Hur YM, Bouchard TJ (1997). The genetic correlation between impulsivity and sensation seeking traits. Behav Genet.

[B34] Fairbanks LA, Newman TK, Bailey JN, Jorgensen MJ, Breidenthal SE, Ophoff RA, Comuzzie AG, Martin LJ, Rogers J (2004). Genetic contributions to social impulsivity and aggressiveness in vervet monkeys. Biol Psychiatry.

[B35] Isles AR, Humby T, Walters E, Wilkinson LS (2004). Common genetic effects on variation in impulsivity and activity in mice. J Neurosci.

[B36] Silverman IW (2003). Gender differences in delay of gratification: A meta-analysis. Sex Roles.

[B37] Dom G, D'Haene P, Hulstijn W, Sabbe B (2006). Impulsivity in abstinent early- and late-onset alcoholics: differences in self-report measures and a discounting task. Addiction.

[B38] Scheres A, Dijkstra M, Ainslie E, Balkan J, Reynolds B, Sonuga-Barke E, Castellanos FX (2006). Temporal and probabilistic discounting of rewards in children and adolescents: Effects of age and ADHD symptoms. Neuropsychologia.

[B39] Godoy R, Byron E, Reyes-Garcia V, Leonard WR, Patel K, Apaza L, Perez E, Vadez V, Wilkie D (2004). Patience in a foraging-horticultural society: A test of competing hypotheses. J Anthropol Res.

[B40] Harrison GW, Lau MI, Williams MB (2002). Estimating individual discount rates in Denmark: A field experiment. Am Econ Rev.

[B41] Olson SL, Bates JE, Sandy JM, Schilling EM (2002). Early developmental precursors of impulsive and inattentive behavior: from infancy to middle childhood. J Child Psychol Psychiatry.

[B42] Preuss UW, Koller G, Bondy B, Bahlmann M, Soyka M (2001). Impulsive traits and 5-HT2A receptor promoter polymorphism in alcohol dependents: Possible association but no influence of personality disorders. Neuropsychobiology.

[B43] Patkar AA, Berrettini WH, Hoehe M, Thornton CC, Gottheil E, Hill K, Weinstein SP (2002). Serotonin transporter polymorphisms and measures of impulsivity, aggression, and sensation seeking among African-American cocaine-dependent individuals. Psychiatry Res.

[B44] Baca-Garcia E, Vaquero C, Diaz-Sastre C, Garcia-Resa E, Saiz-Ruiz J, Fernandez-Piqueras J, de Leon J (2004). Lack of association between the serotonin transporter promoter gene polymorphism and impulsivity or aggressive behavior among suicide attempters and healthy volunteers. Psychiatry Res.

[B45] Retz W, Rosler M, Supprian T, Retz-Junginger P, Thome J (2003). Dopamine D3 receptor gene polymorphism and violent behavior: relation to impulsiveness and ADHD-related psychopathology. J Neural Transm.

[B46] Limosin F, Loze JY, Dubertret C, Gouya L, Ades J, Rouillon F, Gorwood P (2003). Impulsiveness as the intermediate link between the dopamine receptor D2 gene and alcohol dependence. Psychiatric Genetics.

[B47] Cloninger CR (1987). A Systematic Method for Clinical Description and Classification of Personality Variants - a Proposal. Arch Gen Psychiatry.

[B48] Kluger AN, Siegfried Z, Ebstein RP (2002). A meta-analysis of the association between DRD4 polymorphism and novelty seeking. Mol Psychiatry.

[B49] Schinka JA, Letsch EA, Crawford FC (2002). DRD4 and novelty seeking: Results of meta-analyses. Am J Med Genet.

[B50] Savitz JB, Ramesar RS (2004). Genetic variants implicated in personality: A review of the more promising candidates. Am J Med Genet B Neuropsychiatr Genet.

[B51] Nisbett RE, Wilson TD (1977). Telling More Than We Can Know - Verbal Reports on Mental Processes. Psychol Rev.

[B52] Wilson TD, Dunn EW (2004). Self-knowledge: Its limits, value, and potential for improvement. Annu Rev Psychol.

[B53] Evenden JL (1999). Varieties of impulsivity. Psychopharmacology.

[B54] Miller E, Joseph S, Tudway J (2004). Assessing the component structure of four self-report measures of impulsivity. Pers Individ Dif.

[B55] Holt DD, Green L, Myerson J (2003). Is discounting impulsive?. Evidence from temporal and probability discounting in gambling and non-gambling college students. Behav Processes.

[B56] Monterosso J, Ehrman R, Napier KL, O'Brien CP, Childress AR (2001). Three decision-making tasks in cocaine-dependent patients: do they measure the same construct?. Addiction.

[B57] Green L, Fristoe N, Myerson J (1994). Temporal discounting and preference reversals in choice between delayed outcomes. Psychon Bull Rev.

[B58] Logan GD, Dagenbach D, Carr TH (1994). On the ability to inhibit thought and action: a users' guide to the Stop Signal paradigm. Inhibitory processes in attention, memory, and language.

[B59] Cardinal RN, Pennicott DR, Sugathapala CL, Robbins TW, Everitt BJ (2001). Impulsive choice induced in rats by lesions of the nucleus accumbens core. Science.

[B60] Winstanley CA, Theobald DEH, Dalley JW, Cardinal RN, Robbins TW (2006). Double dissociation between serotonergic and dopaminergic modulation of medial prefrontal and orbitofrontal cortex during a test of impulsive choice. Cereb Cortex.

[B61] Cardinal RN, Winstanley CA, Robbins TW, Everitt BJ (2004). Limbic corticostriatal systems and delayed reinforcement. Adolescent Brain Development: Vulnerabilities and Opportunities.

[B62] McClure SM, Laibson DI, Loewenstein G, Cohen JD (2004). Separate neural systems value immediate and delayed monetary rewards. Science.

[B63] Ainslie G (1975). Specious Reward - Behavioral Theory of Impulsiveness and Impulse Control. Psychol Bull.

[B64] Petry NM (2001). Delay discounting of money and alcohol in actively using alcoholics, currently abstinent alcoholics, and controls. Psychopharmacology.

[B65] Vuchinich RE, Simpson CA (1998). Hyperbolic temporal discounting in social drinkers and problem drinkers. Exp Clin Psychopharmacol.

[B66] Bickel WK, Odum AL, Madden GJ (1999). Impulsivity and cigarette smoking: delay discounting in current, never, and ex-smokers. Psychopharmacology.

[B67] Mitchell SH (1999). Measures of impulsivity in cigarette smokers and non-smokers. Psychopharmacology.

[B68] Kirby KN, Petry NM, Bickel WK (1999). Heroin addicts have higher discount rates for delayed rewards than non-drug-using controls. J Exp Psychol Gen.

[B69] Madden CJ, Petry NM, Badger GJ, Bickel WK (1997). Impulsive and self-control choices in opioid-dependent patients and non-drug using control subjects: drug and monetary rewards. Exp Clin Psychopharmacol.

[B70] Coffey SF, Gudleski GD, Saladin ME, Brady KT (2003). Impulsivity and rapid discounting of delayed hypothetical rewards in cocaine-dependent individuals. Exp Clin Psychopharmacol.

[B71] Petry NM (2001). Pathological gamblers, with and without substance use disorders, discount delayed rewards at high rates. J Abnorm Psychol.

[B72] Ohmura YT (2006). Three-month stability of delay and probability discounting measures. Exp Clin Psychopharmacol.

[B73] van Gaalen MM, van Koten R, Schoffelmeer ANM, Vanderschuren L (2006). Critical involvement of dopaminergic neurotransmission in impulsive decision making. Biol Psychiatry.

[B74] Richards JB, Sabol KE, de Wit H (1999). Effects of methamphetamine on the adjusting amount procedure, a model of impulsive behavior in rats. Psychopharmacology.

[B75] Wade TR, de Wit H, Richards JB (2000). Effects of dopaminergic drugs on delayed reward as a measure of impulsive behavior in rats. Psychopharmacology.

[B76] Zuckerman M (1979). Sensation seeking: Beyond the optimal level of arousal.

[B77] Horn NR, Dolan M, Elliott R, Deakin JFW, Woodruff PWR (2003). Response inhibition and impulsivity: an fMRI study. Neuropsychologia.

[B78] Noble EP (1998). The D-2 dopamine receptor gene: A review of association studies in alcoholism and phenotypes. Alcohol.

[B79] Li D, Sham PC, Owen MJ, He L (2006). Meta-analysis shows significant association between dopamine system genes and attention deficit hyperactivity disorder (ADHD). Hum Mol Genet.

[B80] Callier S, Snapyan M, Crom SL, Prou D, Vincent JD, PhilippeVernier (2003). Evolution and cell biology of dopamine receptors in vertebrates. Biol Cell.

[B81] Hartman DS, Lanau F (1997). Diversity of dopamine receptors: new molecular and pharmacological developments. Pol J Pharmacol.

[B82] Oak JN, Oldenhof J, Van Tol HHM (2000). The dopamine D-4 receptor: one decade of research. Eur J Pharmacol.

[B83] Noble EP (2003). D2 dopamine receptor gene in psychiatric and neurologic disorders and its phenotypes. Am J Med Genet B Neuropsychiatr Genet.

[B84] Meador-Woodruff JH, Damask SP, Wang J, Haroutunian V, Davis KL, Watson SJ (1996). Dopamine receptor mRNA expression in human striatum and neocortex.. Neuropsychopharmacology.

[B85] De la Garza R, Madras BK (2000). [H-3]PNU-101958, a D-4 dopamine receptor probe, accumulates in prefrontal cortex and hippocampus of non-human primate brain. Synapse.

[B86] Defagot MC, Antonelli MC (1997). Autoradiographic localization of the putative D4 dopamine receptor in rat brain. Neurochem Res.

[B87] Matsumoto M, Hidaka K, Tada S, Tasaki Y, Yamaguchi T (1996). Low levels of mRNA for dopamine D4 receptor in human cerebral cortex and striatum. J Neurochem.

[B88] Ratsma JE, van der Stelt O, Schoffelmeer ANM, Westerveld A, Gunning WB (2001). P3 event-related potential, dopamine D2 receptor A1 allele, and sensation-seeking in adult children of alcoholics. Alcohol Clin Exp Res.

[B89] Blum K, Braverman E, Holder J, Lubar J, Vincent M, Miller D, Lubar J, Chen T, Comings D (2000). Reward Deficiency Syndrome: A Biogenetic Model for the Diagnosis and Treatment of Impulsive, Addictive and Compulsive Behaviors. J Psychoactive Drugs.

[B90] Thompson J (1997). D2 dopamine receptor gene (DRD2) Taq1 A polymorphism: reduced dopamine D2 receptor binding in the human striatum associated with the A1 allele. Pharmacogenetics.

[B91] Dubertret C, Gouya L, Hanoun N, Deybach JC, Ades J, Hamon M, Gorwood P (2004). The 3 ' region of the DRD2 gene is involved in genetic susceptibility to schizophrenia. Schizophr Res.

[B92] Neville MJ, Johnstone EC, Walton RT (2004). Identification and characterization of ANKK1: A novel kinase gene closely linked to DRD2 on chromosome band 11q23.1. Hum Mutat.

[B93] MacMurray J, Madrid A, Bottini E, Muhleman D, Comings D, Kohler JLRHP (2002). Evidence of an emerging collision between the fertility transition and genotype-dependent fertility differentials. Biodemography of Human Reproduction and Fertility.

[B94] Ding YC, Chi HC, Grady DL, Morishima A, Kidd JR, Kidd KK, Flodman P, Spence MA, Schuck S, Swanson JM, Zhang YP, Moyzis RK (2002). Evidence of positive selection acting at the human dopamine receptor D4 gene locus. Proc Natl Acad Sci U S A.

[B95] Asghari V, Sanyal S, Buchwaldt S, Paterson A, Jovanovic V, Vantol HHM (1995). Modulation of Intracellular Cyclic-Amp Levels by Different Human Dopamine D4 Receptor Variants. J Neurochem.

[B96] Czermak C, Lehofer M, Liebmann PM, Traynor J (2006). [S-35]GTP gamma S binding at the human dopamine D4 receptor variants hD4.2, hD4.4 and hD4.7 following stimulation by dopamine, epinephrine and norepinephrine. Eur J Pharmacol.

[B97] Schoots O, Van Tol HHM (2003). The human dopamine D4 receptor repeat sequences modulate expression. Pharmacogenomics J.

[B98] Van Tol HHM (1992). Multiple dopamine D4 receptor variants in the human population. Nature.

[B99] Van Craenenbroeck K, Clark SD, Cox MJ, Oak JN, Liu F, Van Tol HHM (2005). Folding efficiency is rate-limiting in dopamine D4 receptor biogenesis. J Biol Chem.

[B100] Asghari V, Schoots O, Vankats S, Ohara K, Jovanovic V, Guan HC, Bunzow JR, Petronis A, Vantol HHM (1994). Dopamine D4 Receptor Repeat - Analysis of Different Native and Mutant Forms of the Human and Rat Genes. Mol Pharmacol.

[B101] Oak JN, Lavine N, Tol HHMV (2001). Dopamine D4 and D2L Receptor Stimulation of the Mitogen-Activated Protein Kinase Pathway Is Dependent on trans-Activation of the Platelet-Derivied Growth Factor Receptor. Mol Pharmacol.

[B102] Jovanovic V, Guan HC, Van Tol HHM (1999). Comparative pharmacological and functional analysis of the human dopamine D-4.2 and D-4.10 receptor variants. Pharmacogenetics.

[B103] Watts VJ, Vu MN, Wiens BL, Jovanovic V, Tol HHMV, Neve KA (1999). Short- and long-term heterologous sensitization of adenylate cyclase by D4 dopamine receptors. Psychopharmacology.

[B104] Cho DI, Beorn S, Van Tol HHM, Caron MG, Kim KM (2006). Characterization of the desensitization properties of five dopamine receptor subtypes and alternatively spliced variants of dopamine D-2 and D-4 receptors. Biochem Biophys Res Commun.

[B105] Hamarman S, Fossella J, Ulger C, Brimacombe M, Dermody J (2004). Dopamine receptor 4 (DRD4) 7-repeat allele predicts methylphenidate dose response in children with attention deficit hyperactivity disorder: A pharmacogenetic study. J Child Adolesc Psychopharmacol.

[B106] Brody AL, Mandelkern MA, Olmstead RE, Scheibal D, Hahn E, Shiraga S, Zamora-Paja E, Farahi J, Saxena S, London ED, McCracken JT (2006). Gene variants of brain dopamine pathways and smoking-induced dopamine release in the ventral caudate/nucleus accumbens. Arch Gen Psychiatry.

[B107] Hutchison KE, Wooden A, Swift RM, Smolen A, McGeary J, Adler L, Paris L (2003). Olanzapine reduces craving for alcohol: A DRD4 VNTR polymorphism by pharmacotherapy interaction. Neuropsychopharmacology.

[B108] Hutchison KE, Ray L, Sandman E, Rutter MC, Peters A, Davidson D, Swift R (2006). The effect of olanzapine on craving and alcohol consumption. Neuropsychopharmacology.

[B109] McGough J, McCracken J, Swanson J, Riddle M, Kollins S, Greenhill L, Abikoff H, Davies M, Chuang S, Wigal T, Wigal S, Posner K, Skrobala A, Kastelic E, Ghuman J, Cunningham C, Shigawa S, Moyzis R, Vitiello B (2006). Pharmacogenetics of methylphenidate response in preschoolers with ADHD. J Am Acad Child Adolesc Psychiatry.

[B110] Noble EP, Ozkaragoz TZ, Ritchie TL, Zhang XX, Belin TR, Sparkes RS (1998). D-2 and D-4 dopamine receptor polymorphisms and personality. Am J Med Genet.

[B111] Hutchison KE, McGeary J, Smolen A, Bryan A, Swift RM (2002). The DRD4 VNTR polymorphism moderates craving after alcohol consumption. Health Psychol.

[B112] Hutchison KE, Stallings M, McGeary J, Bryan A (2004). Population stratification in the candidate gene study: Fatal threat or red herring?. Psychol Bull.

[B113] Shields AE, Fortun M, Hammonds EM, King PA, Lerman C, Rapp R, Sullivan PF (2005). The use of race variables in genetic studies of complex traits and the goal of reducing health disparities - A transdisciplinary perspective. Am Psychol.

[B114] GSFL Research Group GSFL Genotyping Protocol - DRD2 (Dopamine Receptor D2) Taq1"A". http://www.chmeds.ac.nz/research/gsfl/drd2taqarflp.pdf.

[B115] Boór K, Rónai Z, Nemoda Z, Gaszner P, Sasvári-Székely M, Guttman A, Kalász. H (2002). Noninvasive Genotyping of Dopamine Receptor D4 (DRD4) Using Nanograms of DNA From Substance-Dependent Patients. Curr Med Chem.

[B116] DeVona J (2005). Delay Discounting Task.

[B117] Mazur JE, Commons ML, Mazur JE, Nevin JA, Rachlin H (1987). An adjusting procedure for studying delayed reinforcement. Quantitative analyses of behavior, The effect of delay and of intervening events on reinforcement value.

[B118] Reynolds B, Schiffbauer R (2004). Measuring state changes in human delay discounting: an experiential discounting task. Behav Process.

[B119] Brzustowski J Hardy-Weinberg Equilibrium Test. http://www2.biology.ualberta.ca/jbrzusto/hwenj.html.

[B120] Wacker J, Reuter M, Hennig J, Stemmler G (2005). Sexually dimorphic link between dopamine D2 receptor gene and neuroticism-anxiety. Neuroreport.

[B121] Perneger TV (1998). What's wrong with Bonferroni adjustments. BMJ.

[B122] Bland M (2000). Multiple significance tests and the Bonferroni correction. An Introduction to Medical Statistics.

[B123] Green L, Myerson J, Ostaszewski P (1999). Discounting of delayed rewards across the life span: age differences in individual discounting functions. Behav Process.

[B124] Kruger D (2004). Sexual selection and the Male:Female Mortality Ratio. Evolutionary Psychology.

[B125] Alessi SM, Petry NM (2003). Pathological gambling severity is associated with impulsivity in a delay discounting procedure. Behav Process.

[B126] Mitchell JM, Fields HL, D’Esposito M, Boettiger CA (2005). Impulsive Responding in Alcoholics. Alcohol Clin Exp Res.

[B127] Reynolds B, Richards JB, Wit H (2006). Acute-alcohol effects on the Experiential Discounting Task (EDT) and a question-based measure of delay discounting. Pharmacol Biochem Behav.

[B128] Erblich J, Lerman C, Self DW, Diaz GA, Bovbjerg DH (2004). Stress-induced cigarette craving: effects of the DRD2 TaqI RFLP and SLC6A3 VNTR polymorphisms. Pharmacogenomics J.

[B129] Erblich J, Lerman C, Self DW, Diaz GA, Bovbjerg DH (2005). Effects of dopamine D2 receptor (DRD2) and transporter (SLC6A3) polymorphisms on smoking cue-induced cigarette craving among African-American smokers. Mol Psychiatry.

[B130] Miller WB, Pasta DJ, MacMurray J, Chiu C, Wu H, Comings DE (1999). Dopamine receptor genes are associated with age at first sexual intercourse. J Biosoc Sci.

[B131] Carrasco X, Rothhammer P, Moraga M, Henriquez H, Chakraborty R, Aboitiz F, Rothhammer F (2006). Genotypic interaction between DRD4 and DAT1 loci is a high risk factor for attention-deficit/hyperactivity disorder in Chilean families. Am J Med Genet B Neuropsychiatr Genet.

