# Supplementary material for: Examining impulsivity as an endophenotype using a behavioral approach: a DRD2 TaqI A and DRD4 48-bp VNTR association study
Source: Behav Brain Funct. 2007 Jan 10;3:2. doi: 10.1186/1744-9081-3-2 (PMC1781951; doi:10.1186/1744-9081-3-2)
Supplement: Additional File 2 — Delay discounting protocol [file 1744-9081-3-2-S2.doc]

**Delay discounting protocol**

The delayed reward was a $100 hypothetical reward to be received in the future compared to 29 reward amounts to be received today; the amounts were as follows: $100, $99.90, $99.50, $99, $96, $92, $85, $80, $75, $70, $65, $60, $55, $50, $45, $40, $35, $30, $25, $20, $15, $10, $8, $6, $4, $2, $1, $0.50, $0.10. Delay time intervals were: 1 week, 2 weeks, 2 months, 6 months, 1 year, 5 years and 25 years. These parameters were based on a previous version of the task [1, 2].

Procedurally, two reciprocal assessments were made. The first used successively decreasing amounts of immediate reward to identify the participant’s point at which they first opted to switch from selecting the discounted reward (e.g., $90) to the delayed full reward ($100) for each increment of time. After the participant switched from choosing the immediate reward, the procedure continued for five additional monetary increments after the participant switched choices to ensure this was indeed the lowest choice. After determining the descending switching point for all seven time intervals, the procedure began again at the first time interval and, starting at the increment five units below the previously determined switching point, successively increased the immediate rewards available until the individual stopped selecting the delayed reward ($100) and returned to accepting the immediate reward. Similarly, the procedure continued for five additional reward levels beyond the switch in decision-making to ensure this point was correctly identified.

To quantify participants’ discounting functions of $100 based on its delay in time, the descending and ascending switching points were averaged for each time interval, creating the point of indifference (POIs) for that interval. The POIs were then employed in the following equation to derive the function used by a participant to discount the rewards [3]:

*V* = *A*/(1 + *kD)*,

where *V* = subjective value of the delayed reward (POI), *A* = full amount of the delayed reward, *k* = empirically determined constant proportional to the degree of delay discounting (i.e., discounting function), and *D* = delay duration. According to this equation, the larger the discounting function (*k*) the individual applies, the more rapidly the value of the reward decreases based on its delay in time, theoretically reflecting greater impulsivity. Nonlinear regression was used to fit the preceding equation and the *R^2^* values were used to determine individual model fits. Erratic subjects and those with *R^2^* values below 0.30 were excluded from principal analyses [4]. Administration of the DDT took approximately 20 minutes.

1. Petry NM: **Delay discounting of money and alcohol in actively using alcoholics, currently abstinent alcoholics, and controls.** *Psychopharmacology* 2001, **154:**243-250.

2. Petry NM: **Discounting of delayed rewards in substance abusers: relationship to antisocial personality disorder.** *Psychopharmacology* 2002, **162:**425-432.

3. Mazur JE: **An adjusting procedure for studying delayed reinforcement.** In *Quantitative analyses of behavior, The effect of delay and of intervening events on reinforcement value.* *Volume* 5. Edited by Commons ML, Mazur JE, Nevin JA, Rachlin H. Hillsdale, NJ: Erlbaum; 1987: 55–73

4. Reynolds B, Schiffbauer R: **Measuring state changes in human delay discounting: an experiential discounting task.** *Behavioural Processes* 2004, **67:**343-356.
