# Supplementary material for: Examining impulsivity as an endophenotype using a behavioral approach: a DRD2 TaqI A and DRD4 48-bp VNTR association study
Source: Behav Brain Funct. 2007 Jan 10;3:2. doi: 10.1186/1744-9081-3-2 (PMC1781951; doi:10.1186/1744-9081-3-2)
Supplement: Additional File 3 — Alternative DRD4 parsing method influences on DRD2 × DRD4 ANOVA to predict impulsivity phenotypes. This supplement explores parsing DRD4 using three alternative methods, and finds similar results to that of the main manuscript. [file 1744-9081-3-2-S3.doc]

**Alternative DRD4 parsing method influences on DRD2 x DRD4 ANOVA to predict impulsivity phenotypes**

**At least one 7R allele versus all others:**

The main effect of DRD2 and DRD2 by DRD4 interaction effects on *k* values remains significant (DRD2: *F*_1,164_= 7.834, *p* =.006 η_p_^2^ =.047; DRD2 x DRD4: *F*_1,164_=7.604, *p* =.007 η_p_^2^ =.045). No other significant genotype by phenotype interactions were found.

**4R/4R subjects versus both 4R/7R and 7R/7R subjects combined:**

A main effect of DRD4 on SSS Experience Seeking was detected such that 7R+ subjects were more Experience Seeking (*F*_1,1135_= 4.305, *p* =.040 η_p_^2^ =.032)

The main effect of DRD2 and DRD2 by DRD4 interaction effects on k values remains significant (DRD2: *F*_1,120_= 8.856, *p* =.004 η_p_^2^ =.071; DRD2 x DRD4: *F*_1,120_=6.526, *p* =.012 η_p_^2^ =.053)

**4R/4Rs subjects versus 4R/7R subjects:**

The main effect of DRD2 and DRD2 by DRD4 interaction effects on k values remains significant (DRD2: *F*_1,117_= 8.259, *p* =.005 η_p_^2^ =.068; DRD2 xDRD4: *F*_1,117_=6.061, *p* =.015 η_p_^2^ =.051). No other significant genotype by phenotype interactions were found.
